# Low SARS‐CoV‐2 seroprevalence in a cohort of Brazilian sickle cell disease patients: Possible effects of emphasis on social isolation for a population initially considered to be at very high risk

**DOI:** 10.1002/jha2.254

**Published:** 2021-06-17

**Authors:** Luiza Francisco Trafane, Vitor Antonio da Costa, Adriana da Silva Santos Duarte, Audrey Basso Zangirolami, José Luiz Proenca‐Modena, Paula de Melo Campos, Samuel de Souza Medina, Sara Terezinha Olalla Saad, Marcelo Addas‐Carvalho, Bruno Deltreggia Benites

**Affiliations:** ^1^ Hematology and Transfusion Medicine Center University of Campinas Campinas Brazil; ^2^ Laboratory of Emerging Viruses (LEVE), Department of Genetics, Evolution, Microbiology and Immunology, Institute of Biology University of Campinas Campinas Brazil

## Abstract

Despite being initially considered at higher risk for severe COVID‐19, sickle cell disease (SCD) patients have mostly presented clinical severity similar to the general population. As their vulnerability to become infected remains uncertain, we assessed the seroreactivity for SARS‐CoV‐2 to estimate the prevalence of infection and possible phenotypic and socioeconomic determinants for their contagion. Serologic evaluation was performed on 135 patients with an overall prevalence of 11%; positivity was associated with older age and use of public transportation. We speculate that social distancing instructions recommended by our clinic may have contributed to lower levels of infection, but potential protection factors need further investigation.

## 1 INTRODUCTION

The high spread rate of the new coronavirus (SARS‐CoV‐2) has imposed massive challenges to health systems, especially considering the high potential of aggravation and mortality in specific populations, such as in the elderly and bearers of comorbidities [1]. In this context, patients with sickle cell disease (SCD) were expected to be at high risk for severe COVID‐19, considering the damage in target organs such as the lungs and kidneys [2], which are also targeted in SARS‐CoV‐2 pathophysiology [3].

SARS‐CoV‐2 infection has been described to precipitate vaso‐occlusive crises in SCD and there is a crescent number of studies assessing the impact of COVID‐19 on SCD patients, but with contradictory results regarding morbidity and mortality [4,5]. In fact, it is interesting to highlight that the series of cases published so far show an unexpected trend to milder presentations in SCD, with high full‐recovery rates [6–8].

Nevertheless, it is not yet possible to establish whether this mild picture is the standard to be observed in geographic locations other than the developed countries, such as Latin America and Africa, where the socioeconomic conditions and the access to health services tend to be precarious and can have a major impact on both the exposure to COVID‐19 and the treatment of the basal condition. In face of these challenges, our service established protocol changes to minimize patient contact with the nosocomial environment and to favor social distancing, while maintaining the best possible assistance regarding both chronic and emergency care.

Thus, this study aimed to analyze the trend of seroreactivity for SARS‐CoV‐2 in SCD patients in a Brazilian Center and its distribution over time (August 2020 to January 2021), as well as its correlations with clinical and sociodemographic variables and the degree of social isolation actually achieved by these individuals.

## 2 MATERIALS AND METHODS

### 2.1 Study population, questionnaire, and blood sampling

The study was conducted at the Hematology and Transfusion Medicine Center of the University of Campinas, a public reference center for the care of SCD in southeastern Brazil. Patients with any genotypic presentation (HbSS, Sβ‐thalassemia, or HbSC) and above 18 years of age were included. The study was approved by the Ethics Committee of the hospital (approval number 4.153.469).

Clinical data were recorded, including comorbidities, medications, the presence of symptoms suggestive of SARS‐CoV‐2 infection, and SCD complications since March/2020 (when the first COVID‐19 case was reported in Brazil), including the need for hospital admission. Socioeconomic evaluation included the assessment of the level of social isolation achieved in the period and perceptions regarding its effectiveness. The collection of blood for serologic tests was repeated at each visit during a 6‐month period (August 2020 to January 2021) to characterize the dynamics of seroprevalence and possible seroconversions. In the same period, a serological survey was carried out in a sample of 2200 blood donors in the center in the same period of time, in order to compare the prevalence in a healthy population with sociodemographic equivalence.

### 2.2 Laboratory tests

Samples were screened by chemiluminescence (reactivity considered positive: IgG ≥1.4 and IgM ≥1.0), according to the manufacturer's instructions (Abbott Architect, Ireland). Reagent samples were tested for the presence of neutralizing antibodies and their titration, as previously described [9]. After 3 days of incubation of Vero cells with patient serum–virus mixture, cells were inspected and the highest serum dilution that protected cells from cytopathic effect was taken as the neutralization titer.

### 2.3 Statistical analysis

Data are reported by frequency measures, and correlations of variables with positivity in serological tests were assessed using Fisher's exact test or *t*‐test for independent samples.

## 3 RESULTS

### 3.1 Patients’ clinical characteristics and test results

**FIGURE 1:**
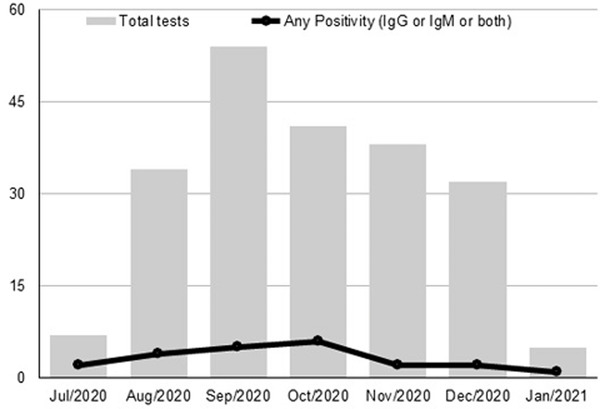
Temporal distribution of serological tests and positive results

In all, 214 tests were performed on 135 patients (86% of SCD patients registered at the center) from August 2020 to January 2021 (Figure 1), including 82 HbSS (61%), 41 HbSC (30%), eight Sβ+ (6%), and four Sβ0 patients (3%); 57% were male, with median age of 42 years (19–74), and 66% were using hydroxyurea. During the analyzed period, 61 (45%) patients had vaso‐occlusive crises; 37 (27%) had symptoms suggestive of COVID‐19, but only two patients had a positive PCR for SARS‐CoV‐2 when evaluated (Table 1). Unfortunately, it was not possible to access which nor how many of the SARS‐CoV‐2 variants were involved in our patients’ infections.

**TABLE 1:** General patients’ clinical and demographic characteristics

	All patients (*n* = 135)
Median age (range), years	42 (19–74)
Median family income, USD/year	$13,090.9
Median personal income, USD/year	$4363.64
Male:female, *n*	77:58
Genotype (*n*): SS:SC:Sβ+:Sβ0	82:41:8:4
Use of hydroxyurea, *n*	89 (66%)
Chronic transfusion, *n*	5 (4%)
Vaso‐oclusive crisis, *n*	61 (45%)
COVID‐19 symptoms, *n*	37 (27%)

Regarding serological evaluation, 57 patients (42%) were tested more than once during this period: 46 with two tests, 11 with ≥3 tests. Among all patients, 15 had positive results: nine IgG and IgM+ and six IgG+ only; therefore, with an overall seroprevalence of 11% (despite 27% of them having reported some symptom of COVID‐19 during the analyzed period). Seroconversion was documented for only one patient and, interestingly, with no signs or symptoms of infection. Moreover, two patients lost IgG positivity 3 months after the initial positive test. The search for functionally neutralizing antibodies resulted in nine patients with low titers (<1:40) and only three patients with remarkably high titers (≥1:640).

When asked about previously diagnosed comorbidities, among the 116 patients that responded to the questionnaire, 16% reported a diagnosis of hypertension, 14% were obese (according to BMI calculated according to reported weight and height), and 15% were diabetic. Besides that 2.6% of patients self‐declared as bearers of chronic kidney disease (CKD) and 2.6% reported being asthmatic. When questioned about vaso‐occlusive crisis in the period between March of 2020 (first reported coronavirus case in the country) and the application of the questionnaire, 45% of patients declared an SCD‐related pain crisis, and 13% of these patients required hospitalization for medical assistance due to the severity of pain. Among all patients, 17.2% had a history of previous acute chest syndrome (ACS) and 11.2% had persistent skin ulcers. Among the 15 patients with positive serologies for COVID‐19 antibodies, five were hypertense, three were obese, one had chronic kidney disease, two had a previous stroke, one had asthma, and one was diagnosed with Chagas disease alongside the SCD. Four of the patients positive for COVID‐19 had a history of persistent skin ulcers and three had previous ACS episodes. Only four of the COVID‐19‐positive patients declared not having any comorbidities or SCD‐related complications.

### 3.2 Individual perception of pandemic

When questioned, only 12% of patients did not consider themselves vulnerable to infection and only 17% believed that individual and collective protection measures were expendable. In fact, 91% stated that they were able to adopt social distance measures: 76% reported cancelation of social events and 64% managed to reduce the use of public transport, however only 44% could work or study remotely.

### 3.3 Protocol adaptations for care of SCD patients during COVID‐19 pandemic and their possible impact on susceptibility

Social isolation of patients with hemoglobinopathies was recommended by our clinic, in line with national and international guidelines, considering that these patients could potentially develop severe SARS‐CoV‐2 infection. All routine appointments were canceled, and patients were encouraged to contact the center by phone or email at any time in case of whichever doubts they might have. Prescriptions for hydroxyurea, which are withdrawn at the public system, had their renewal period extended from 3 to 6 months. In addition, the center adopted a COVID‐19 suspicion protocol at the entrance, avoiding the access of any individual with the slightest suspicion of infection or who had been in contact with a person presenting symptoms; those who presented symptoms were referred to a specific COVID‐19 unit at the university hospital.

Appointments were maintained when the issue could not be solved over the phone: 65% of them comprised symptoms that required personalized assessment or laboratory propedeutic, followed by the evaluation for symptoms of worsening anemia (17%) or re‐evaluation after recent hospital discharge (18%).

Regarding patients requiring hospitalization, either health care took place at the city of origin under the guidance of a hematologist by phone, or patients were attended at the university hospital. A total of 18 patients required hospital admission from March 2020 to January 2021, comprising 14 HbSS, one Sβ+, and three HbSC patients, being 10 females and eight males, with a median age of 41 years (25–74). The search for medical care occurred due to several different symptoms (pain, uncontrolled hypertension, hemiplegia, diarrhea), with three patients admitted with dyspnea and two patients requiring oxygen supplementation. Only five of these patients were tested for COVID‐19 and only two tested positive using a nasopharyngeal swab RT‐PCR. Only one patient (male, HbSS, 49 years old, complications: bilateral leg ulcers and hepatic iron overload) needed hospitalization specifically for COVID‐19 complications, including the need for mechanical ventilation and exchange transfusions, but was discharged after 14 days. There were no deaths during the study period and the average length of hospital stay was 6.25 days.

In this study's cohort, there were no correlations between serological positivity and education, income, number of household contacts, and maintenance of work outside the home; however, test positivity was associated with older age (40.3 × 22.9, *p* < .001) and regular use of public transport (Fisher exact test, *p* = .02).

## 4 DISCUSSION

This report highlights the importance of new measures of distant medical assistance for SCD in the context of a pandemic, which depends on the commitment of the medical team and confidence of the patients in this team. Therefore, interesting approaches have emerged, such as less bureaucracy in the dispensation of hydroxyurea and the occupation of secondary hospital beds that were not referenced for COVID assistance, for more immediate and personalized care. All these measures, taken together, provided less exposure for patients and maximized their social isolation, certainly contributing to a lower vulnerability to SARS‐CoV‐2 infection than initially projected. Therefore, we must emphasize that the lower availability of population testing in our country no doubt leads to a lower acknowledgment of cases. However, despite this fact, we were able to demonstrate that some patients had COVID‐19 in such a benign form that they did not seek medical attention and infection was only revealed by retrospective serological evaluation.

The prevalence of anti‐SARS‐CoV‐2 antibodies was accessed in our center's blood donors in the same period (data yet not published), and we could verify an overall prevalence of 10.5% in this population. The blood donor pool comes from the same geographic area and is, therefore, a comparable parameter. The fact that there was little difference between the seroprevalence of antibodies between a population apt for blood donation (hence healthy individuals) and an SCD patient cohort with high rates of comorbidities suggests that the social distancing instructions given to patients by our SCD clinic throughout the pandemic may have contributed to lower levels of SARS‐CoV‐2 infection in our cohort, given the patients’ compliance to social distancing measures attested by the surveys. The fact that only one patient at our center had severe disease during the study period agrees with other reports showing less severity for SCD than initially expected [10], which raises the question regarding protection factors in these patients, or at least may rule out an increased risk for severe disease in this specific population. In fact, the transient positivity of serological tests and the low levels of functional neutralizing antibodies in SCD patients may indicate the acquisition of protective immune responses that are not dependent on antibodies and that remain to be better evaluated.

This is the first article that accesses seroprevalence of anti‐SARS‐CoV‐2 antibodies in SCD population. Therefore, we do not have parameters to access comparatively the pattern of antibody titers and duration of serologic positivity in this specific population. Studies in the general population have attested that, even in a broader spectrum of patients, the duration of antibody rise is still not completely elucidated [11] and more research is imperative in this area.

It can be questioned if hydroxyurea could have been a protective factor against COVID‐19 infection, since 66% of patients were under therapy with the drug. Among the overall 15 patients with positive results for COVID‐19 antibodies, 10 were in use of hydroxyurea. In fact, Minniti et al. [10] have pointed hydroxyurea use as a protective factor against death in their cohort of SCD patients with COVID‐19.

Unfortunately, subsequently to the end of the study in January 2021, there was an upsurge of the pandemic in the country, with high prevalence of the P1 variant. Despite not having the serologic results of the patients in the period between January and April, only one patient was hospitalized due to COVID‐19 diagnosis in this period. The patient was a 56‐year‐old male with a critical phenotype (repeating acute chest syndrome episodes) and hypertension. He progressed to death after 3 weeks of mechanical ventilation. Therefore, we understand that even with the new variant, notwithstanding specific serological evaluation, there has not been a significant increase in our patients’ infection rates or disease severity.
